# The Role of CD44 in the Pathophysiology of Chronic Lymphocytic Leukemia

**DOI:** 10.3389/fimmu.2015.00177

**Published:** 2015-04-20

**Authors:** Julia Christine Gutjahr, Richard Greil, Tanja Nicole Hartmann

**Affiliations:** ^1^Laboratory for Immunological and Molecular Cancer Research, 3rd Medical Department with Hematology, Medical Oncology, Hemostaseology, Infectiology and Rheumatology, Oncologic Centre, Paracelsus Medical University, Salzburg, Austria; ^2^Salzburg Cancer Research Institute, Salzburg, Austria

**Keywords:** CD44, chronic lymphocytic leukemia, microenvironment, homing, hyaluronan

## Abstract

CD44 interactions with hyaluronan (HA) play a key role in various malignancies, supporting tumor cell migration, adhesion, and survival. In contrast to solid tumors, the expression of CD44 standard and variant forms and their functional interplay with HA is less understood in hematological malignancies. Chronic lymphocytic leukemia (CLL) is a highly abundant B-cell malignancy with a well coordinated balance between cell cycle-arrest and proliferation of tumor subpopulations. The long-term survival and proliferation of CLL cells requires their dynamic interactions with stromal and immune cells in lymphoid organs. Interactions of HA with CD44 and HA-mediated motility receptor (RHAMM) contribute to CLL cell localization, and hence CLL pathophysiology, by shaping homing, interstitial migration, and adhesion of the tumor cells. CD44 can complex with key prognostic factors of CLL, particularly CD38 and CD49d, bridging the gap between prognosis and cellular function. Here, we review the current evidence for the individual and associated contributions of CD44 to CLL pathophysiology, the dynamic functional regulation of CD44 upon CLL cell activation, and possible therapeutic strategies targeting CD44 in CLL.

## Introduction

The tumor microenvironment, shaped by interactions between malignant and non-malignant cells, is influential for tumor formation and progression of various cancers. Chronic lymphocytic leukemia (CLL) is a disease of mature B lymphocytes and is manifested by progressive accumulation of these malignant cells in blood, bone marrow (BM), and lymphoid tissues (1). Characteristically, CLL follows extremely variable clinical courses with survival times ranging from months to decades, making it necessary to classify the patients according to prognostic risk (2). Besides genomic aberrations such as 17p deletion, 13q deletion, trisomy 12, and 11q deletion, a most important and long-established prognostic marker is the mutational status of the B-cell receptor (BCR) immunoglobulin variable heavy chain (IgVH) genes (2–4). Patients with CLL cells that express IgVH genes without significant levels of mutation (<2% difference from germline gene counterpart, “unmutated”) follow a more aggressive clinical course with shorter times to first treatment and overall survival than patients harboring IgVH gene mutations (≥2% difference from germline, “mutated”) (5). Other common prognostic parameters are the extent of expression of CD38 and zeta-chain-associated protein kinase 70 (ZAP-70), earlier suggested as surrogate markers for the IgVH mutation status (6). However, both have independent prognostic power, too.

CD49d, despite being the newest among the prognostic markers, is the strongest flow cytometry-based predictor of overall survival and treatment-free survival in CLL (7). Following the first reports on the poor outcome of patients with an expression of CD49d on ≥30% of the tumor cells (8, 9), its high prognostic relevance has been unequivocally confirmed by several groups (10–15). Expression of CD38 and CD49d is associated in about 80% of samples (12, 16) and the molecules can form macromolecular complexes with CD44 (17, 18).

It is well established that the CLL pathophysiology relies on the lymphoid tumor microenvironment. Unusual for tumor cells, CLL cells circulating in the peripheral blood are cell cycle arrested. *Ex vivo*, CLL cells rapidly die from apoptosis if not co-cultured with immune or stromal cells, suggesting that the malignant cells are in constant need of supportive signals from the lymphoid microenvironment (19). It is therefore believed that at least a subpopulation of the peripheral blood CLL pool is able to recirculate into lymphoid organs in order to receive signals for proliferation and survival. Moreover, retention in these organs appears to favor onset and progression of CLL. Consequently, therapeutic targeting the microenvironmental interactions and lymphoid localization of the malignant cells emerges as a most successful strategy to permanently control CLL. This is impressively reflected by the clinical success of novel drugs such as ibrutinib and idelalisib that inhibit downstream signals of the BCR and retention molecules (20–23). Notably, the mode of action of ibrutinib and idelalisib is likely dual, they antagonize tumor cell proliferation in a NF-κB dependent manner (24, 25) and disrupt CLL cell retention in lymphoid organs. Particularly, during the first period of treatment with these drugs, a redistribution of CLL cells from the lymphoid organs into the peripheral blood of patients can be observed (21, 26, 27), obviously depriving the tumor cells of supportive signals.

Despite this recent therapeutic progress, the detailed mechanisms that underlie the communication of CLL cells and accessory cells in the lymphoid microenvironment are still far from understood. Adhesion molecules and homing receptors orchestrate the localization and retention of CLL cells in lymphoid proliferation areas where CLL cells receive activation and protection signals. The glycoprotein CD44 can direct microenvironmental communication and intracellular signaling for growth and motility in many types of cancers (28). On hematopoietic cells, CD44 is universally expressed (28). The CD44 gene encodes different CD44 variant (CD44v) isoforms, which are generated by alternative splicing. The standard isoform of CD44 (CD44s) lacks the entire variable region. Hyaluronan (HA), the main ligand of CD44, is bound via a conserved BX_7_B binding motif (in which B represents Arg or Lys and X7 represents any seven non-acidic amino acids, but includes an additional Arg or Lys) present in the extracellular part of CD44 (28). The binding ability of the ubiquitously expressed molecules CD44 and HA needs to be strictly controlled. This can be achieved by posttranslational modifications such as glycosylations, CD44v expression, or CD44 clustering (28). In CLL, an external activation stimulus leads to increased CD44v expression and N-linked glycosylation, which induces CD44–HA binding (29). Concordantly, many studies have implicated CD44v rather than CD44s in tumor progression, dependent on the stage of progression and type of tumor (28).

In CLL, elevated CD44s and CD44v serum levels have been suggested as markers for disease progression and potential functional contributions to the pathophysiology have been discussed; however, the underlying biological mechanisms remain elusive. With some aspects controversially described, it has become necessary to further examine and more deeply understand the role of CD44 in this disease. Here, we discuss the prognostic role of CD44 and CD44v, its involvement in localization of CLL cells in lymphoid organs and tumor cell survival, and its suitability for therapeutic exploitation.

## HA Receptors and CLL Prognosis

CD44 is described to form a complex with the prognostic markers CD49d and CD38, outlined in the introduction (18, 30). However, first reports on an individual prognostic role of CD44 in CLL were already published in the early 1990s (31), long before this complex was found. Despite this early discovery, the existing data are not completely consistent. In 1993, de Rossi and colleagues distinguished three groups of CLL patients, depending on either high, intermediate or low CD44 surface expression, defined in relation to the CD44 expression on T-cells. In this study, patients of the CD44-high group presented with an increased incidence of diffuse BM infiltration, which is a negative prognostic marker itself (31–33). Illogically, the follow up study of the same group identified these CD44-intermediate/high classified patients as good clinical outcomes (34). Subsequently and more consistent to the early findings, Eisterer and colleagues confirmed the prognostic value of CD44 by immunohistochemistry of BM specimen. CD44-high patients presented with advanced disease, a diffuse pattern of BM infiltration, and reduced survival within the observation period (35). Much later, Herishanu et al. (36) suggested that IgVH unmutated CLL cases express higher CD44 expression (36). We did not find any differences in the intensity of CD44s expression in low and high risk patients, stratified according to IgVH mutation status, CD38, ZAP-70, or CD49d expression (29). This was confirmed by Fedorchenko et al. (37) when grouping patients according to IgVH mutational status or ZAP-70 expression.

The reason of these diverging observations remains unclear but one could hypothesize a differential activation status of the samples. We found that CD44 surface expression of CLL cells is induced upon their stimulation with activated T-cells or CD40 Ligand (CD40L) (29). In addition, several variant isoforms of CD44, known as markers for tumor progression in various malignancies (28), are transcribed and expressed at the surface upon activation (29).

In resting CLL cells, however, surface expression of CD44v is only detectable in the minority of CLL cases (38). These cases differ from the CD44v low expressing cases in regard to disease progression, lymphocyte doubling time, and therapy requirement (39). We found transcripts of CD44v3, v5, v6, v7, v8, v9, and v10 in unstimulated CLL cells, and a robust upregulation of CD44v3 and v6 upon CLL cell activation (29).

Soluble CD44, lacking the transmembrane region (40, 41) is found in serum due to shedding events (40, 42). High serum levels of CD44s, elevated in approximately half of CLL samples, are significantly associated with high tumor burden and the presence of other unfavorable prognostic markers such as high beta2-microglobulin levels (38, 43). The correlation is stable in time, treatment independent, and allows separation of two distinct patient groups with differential survival times (38). While de Rossi and colleagues did not observe any differences in CD44v in serum of CLL patients compared to healthy donors (38), a later study by Eisterer and colleagues identified elevated serum CD44v6 levels being associated with advanced disease defined by lymph node involvement and splenomegaly, and therapy requirement (44). This divergence was attributed to differential sensitivities of the statistical tests used. Nevertheless, independent analyses are required to solve these issues, particularly in case of CD44v6.

In CLL, little is known on the role of the second major HA-binding molecule RHAMM. One report describes a prominent expression of RHAMM and its splice variant RHAMM^−exon 4^ in advanced CLL (45). As RHAMM expression was missing in peripheral blood mononuclear cells (PBMCs) from healthy individuals, it was suggested as a tumor-associated antigen (TAA) in CLL (45). A follow up study provided evidence of an additional prognostic role of RHAMM expression among CLL patients with mutated IgVH genes (46).

## Migration and Localization

The CD44 molecule was originally defined as a lymphocyte homing receptor that can be bound by the Hermes class of antibodies (47–49). Homing hereby means the rapid process, in which circulating hematopoietic cells actively cross the blood/endothelium barrier to enter the tissue (50). BM homing of normal progenitor cells is dependent on CD44 expressed on these cells and HA displayed on the BM endothelium (51–53). Moreover, CD44 participates in homing and engraftment of various tumor cells (54–57).

The contribution of CD44 to homing of CLL cells to BM and secondary lymphoid organs has not been dissected yet. However, we have previously established the integrin VLA-4, a heterodimer of the negative prognostic marker CD49d and the beta1 integrin subunit CD29, as the chief orchestrator of CLL BM homing (12, 58). Moreover, it was also shown that interaction of E-selectin with a specific glycoform of CD44 (HCELL) induces VCAM-1 binding of VLA-4. Thereby, HCELL ligation triggers inside-out upregulation of VLA-4 adhesiveness via G-protein dependent signal transduction leading to firm adhesion and subsequent transendothelial migration of human mesenchymal stem cells (59). Notably, in CLL, CD44v and VLA-4 constitute a cell surface docking complex for matrix metalloproteinase 9 (MMP-9) (in the pro and active form) (30). Here, proMMP-9 does not act as a protease upon docking to this surface receptor complex but fulfills functions in promoting CLL cell survival (60). MMP-9 lacks a transmembrane domain and is therefore dependent on cellular binding sites for all directed functions (61). Most recently, it was observed that high proMMP-9 expression and binding to these sites inhibits migration and reduces the homing capacity of CLL cells, suggesting a cooperation of VLA-4 and CD44(v) with MMP-9 (in the pro and active form) leading to CLL cell retention in lymphoid organs (62).

**Figure 1:**
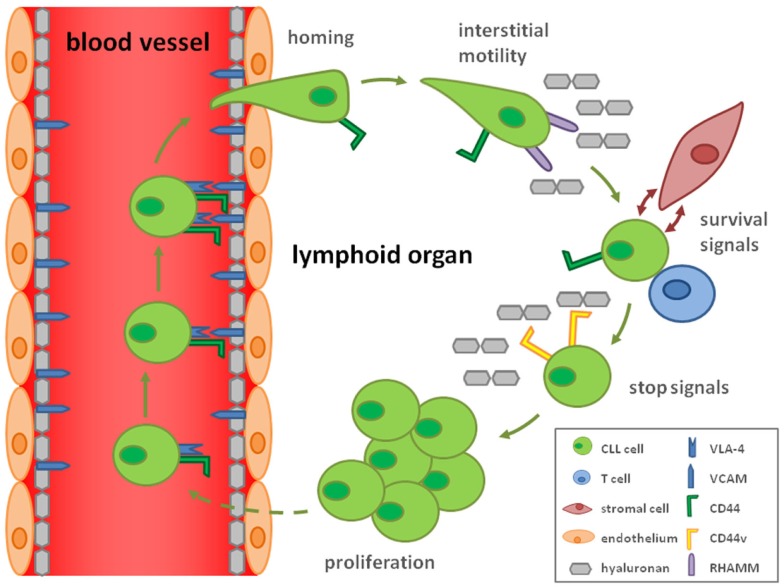
**Hypothetical model how CD44 and CD44v contribute to the CLL cell life cycle**. By the ability of CD44 to complex with VLA-4 (CD49d/CD29), a key molecule for homing of CLL cells, CD44 may influence the homing process. Interstitial migration in the lymphoid organs is CD44 independent but mediated by RHAMM binding to hyaluronan. Interactions with T-cells and hyaluronan-displaying stromal cells secure CLL cell survival and activate the malignant cells. Activation is responsible for a rearrangement from CD44s to CD44v expression enhancing the affinity for hyaluronan, which induces a stop signal for the CLL cell. This retention allows CLL cell proliferation.

Consistent with this idea of CD44-mediated stop signals, we discovered that upon CLL cell activation by T-cells in lymphoid organs, high avidity CD44–HA interactions are formed due to induction of CD44v, most prominently CD44v6, harboring N-linked glycosylations. These interactions result in reduced cellular motility and lock CLL cells to immobilized HA. Thus, activation results in stop signals to migrating CLL cells by inducing strong cellular adhesion to the substrate, which may subsequently allow proliferation (29) (Figure 1). Since MMP-9 is particularly bound to CD44v rather than CD44 (30), it will be interesting how the suggested functions of MMP-9 in CLL are modulated by the activation-induced CD44v expression (29) and contribute to proliferation.

The suggestion of CD44(v) as a retention signal of interstitial motility – a process completely different from homing – is in line with early reports on the involvement of RHAMM rather than CD44 in IL-8-triggered motility of CLL cells on HA (63). This is consistent to our findings that blocking CD44 does not interfere with motility of resting CLL cells under shear free conditions but antagonizes their HA binding and adhesion, once they are activated (29).

## CD44 and CLL Cell Survival

Human CD44 expression is increased by microenvironmental stimulation of CLL cells not only by CD40L-induced activation (29, 37) but also by the presence of feeder cells, known to provide prosurvival signals and early activation (64, 65). Activated CLL cells are protected against spontaneous and drug-induced apoptosis (66–68). Several previous studies suggested that CD44 is part of the survival signaling in CLL (37, 64, 69, 70). The addition of blocking anti-CD44 antibodies to CLL co-cultures with follicular dendritic cells reduced the survival of CLL cells, paralleled by decreased levels of the anti-apoptotic protein myeloid cell leukemia sequence 1 (Mcl-1) (64). Recently, Federochenko and colleagues recapitulated the inhibitory effect of CD44 blockage or downregulation on Mcl1 protein levels *in vitro* and *in vivo*. To study the impact of CD44 on murine leukemogenesis, the authors used CD44 gene deletion, crossing CD44^−/−^ animals with Eμ-TCL1 transgenic (tg) mice (37), which represent a well established murine model for CLL (71). In these mice, a CD5/CD19-double positive clonal B-cell hyperplasia arises in the peritoneal cavity and the disease subsequently spreads into other organs (spleen, BM, LNs, blood), with an overt leukemic phase starting from 8 to 10 months (71, 72). Eμ-TCL1 tg CD44^−/−^ mice displayed a reduced peripheral blood tumor load at 12 months and significantly reduced spleen weights (37) compared to Eμ-TCL1 tg CD44^+/+^ mice. The CD44 deficient murine CLL cells had marked signs of apoptosis, e.g., increased expression of cleaved caspase-3, suggesting a role of CD44 in tumor cell survival in the spleen microenvironment.

Notably, a novel humanized anti-CD44 mAb, RG7356, was recently found to induce apoptosis particularly in ZAP-70 positive CLL cells, in a caspase-dependent manner (70). The effects of this antibody occurred independent of complement and immune-effector cells and were attributed to ligation of CD44, altering its potential complexing with ZAP-70. This involvement of ZAP-70 in CD44-mediated CLL survival signaling and its physical complexing with CD44 clearly impacts on BCR signaling. ZAP-70 is known as an enhancer of BCR signaling upstream of survival and proliferation signals such as protein kinase B (Akt) and extracellular-signal-regulated kinases (ERKs) (73), which in turn induce anti-apoptotic proteins such as Mcl-1 and Bcl-xL (74). In consequence, the data may also suggest alterations of the known complex of MMP-9 with CD44 and CD49d (VLA-4) (60) dependent on the BCR reactivity, which is shaped by ZAP-70.

## Therapy

Therapeutically, CD44 is difficult to exploit due to its high variability and ability to complex with different partners in which CD44 function is apparently influenced. It is therefore not surprising that CD44 is not easily druggable, with some cases of previous failures (clinical trial identifier: NCT02254031; NCT02254044). The anti-CD44v6 antibody bivatuzumab (previously BIWA 4) coupled with a non-radioactive cytotoxic drug mertansine, for example, was used in studies against breast neoplasms (NCT02254005) and squamous cell carcinomas of the head and neck (HNSCC) (NCT02254018) (75–77). The death of one patient terminated the HNSCC trial (NCT02254044) whereas in the breast cancer study the antibody was found in non-tumor tissue as well and was therefore stopped (NCT02254031).

Nevertheless, several recent approaches could be advanced from preclinical status to testing in clinical trials. One promising candidate is the Å6 peptide (Ac-KPSSPPEE-amide), which is derived from the non-receptor binding domain of urokinase plasminogen activator and known to share a homologous sequence with CD44 (78, 79) (see also article by Finlayson in this volume). This homologous sequence (120-NASAPPEE-127) is found in the HA-binding site and is therefore present in all CD44 isoforms independent of alternative splicing events (80, 81). In preclinical studies, treatment with the Å6 peptide significantly decreased tumor growth and metastasis in a breast cancer mouse model without direct evidence of cytotoxicity or anti-proliferative activities toward the tumor (78). Instead, tumor and endothelial cell migration was clearly impaired by the peptide suggesting its impact on tumor invasion, metastasis, and angiogenesis. Similarly, Å6 reduced lymph node metastasis in a prostate cancer model (82). Notably, Å6 inhibited the migration of a subset of ovarian and breast cancer cell lines *in vitro* by inducing high adhesion of the CD44-expressing cells to an HA substrate and altering CD44 conformation (79), obviously locking the cells to substrates of HA, abundantly found, e.g., in LNs. First clinical trials demonstrated that Å6 was well tolerated (83, 84), resulting at least in an increased time to clinical disease progression of women with epithelial ovarian, fallopian tube, or primary peritoneal cancer in clinical remission (85) (NCT00083928). Currently a phase 2 trial is under way to determine the safety, tolerability, and efficacy of Å6 in CLL patients (NCT02046928).

A second promising candidate in CLL is the anti-CD44 antibody RG7356 (also known as RO5429083 or ARH460-16-2), a humanized antibody targeting a glycosylated, extracellular constant region of CD44 (86). As outlined above, this apoptosis-inducing antibody exerts a particular influence on BCR signaling in CLL and may be promising in light of the current success of all BCR-downstream-signal-targeting drugs.

Two clinical trials are underway to examine the pharmacokinetics, pharmacodynamics, safety, and efficacy of RG7356 in acute myelogenous leukemia (AML) patients and patients with metastatic and/or locally advanced CD44-expressing solid tumors.

## Summary and Open Questions

In summary, CD44 emerges as a key molecule of CLL cell interactions with the lymphoid microenvironment, shaping malignant cell positioning, and in consequence survival and proliferation in a fine-tuned manner. Nevertheless, some open questions remain on the mode of CD44 regulation in dependence of the activation status of the cells and the respective complex partner, such as CD49d/VLA-4. In addition, little is known on the second chief receptor interacting with HA, RHAMM. It is conceivable that RHAMM and CD44 fulfill distinct functions of cell migration and retention in CLL, which should be addressed in more detail in future. A deeper understanding of the functional regulation of CD44–HA interactions by splicing events and posttranslational modifications might help solving the existing controversies of its role in prognosis and survival. More functional studies as well as comprehensive patient cohorts and a clear clinical stratification of the patient groups would allow addressing these issues with sufficient statistical power and also assisting in the choice of the appropriate type of CD44 antagonizing therapy in CLL.

## Conflict of Interest Statement

The authors declare that the research was conducted in the absence of any commercial or financial relationships that could be construed as a potential conflict of interest.

## References

[1] Dighiero G Hamblin TJ. Chronic lymphocytic leukaemia. Lancet (2008) 371(9617):1017–29. 10.1016/S0140-6736(08)60456-0 18358929

[2] Damle RN Wasil T Fais F Ghiotto F Valetto A Allen SL Ig V gene mutation status and CD38 expression as novel prognostic indicators in chronic lymphocytic leukemia. Blood (1999) 94(6):1840–7. 10477712

[3] Dohner H Stilgenbauer S Benner A Leupolt E Krober A Bullinger L Genomic aberrations and survival in chronic lymphocytic leukemia. N Engl J Med (2000) 343(26):1910–6. 10.1056/NEJM200012283432602 11136261

[4] Hamblin TJ Davis Z Gardiner A Oscier DG Stevenson FK. Unmutated Ig V-H genes are associated with a more aggressive form of chronic lymphocytic leukemia. Blood (1999) 94(6):1848–54. 10477713

[5] Chiorazzi N Ferrarini M. B cell chronic lymphocytic leukemia: lessons learned from studies of the B cell antigen receptor. Annu Rev Immunol (2003) 21:841–94. 10.1146/annurev.immunol.21.120601.141018 12615894

[6] Crespo M Bosch F Villamor N Bellosillo B Colomer D Rozman M ZAP-70 expression as a surrogate for immunoglobulin-variable-region mutations in chronic lymphocytic leukemia. N Engl J Med (2003) 348(18):1764–75. 10.1056/NEJMoa023143 12724482

[7] Bulian P Shanafelt TD Fegan C Zucchetto A Cro L Nuckel H CD49d is the strongest flow cytometry-based predictor of overall survival in chronic lymphocytic leukemia. J Clin Oncol (2014) 32(9):897. 10.1200/JCO.2013.50.8515 24516016 PMC4876311

[8] Gattei V Bulian P Del Principe MI Zucchetto A Maurillo L Buccisano F Relevance of CD49d protein expression as overall survival and progressive disease prognosticator in chronic lymphocytic leukemia. Blood (2008) 111(2):865–73. 10.1182/blood-2007-05-092486 17959854

[9] Shanafelt TD Geyer SM Bone ND Tschumper RC Witzig TE Nowakowski GS CD49d expression is an independent predictor of overall survival in patients with chronic lymphocytic leukaemia: a prognostic parameter with therapeutic potential. Br J Haematol (2008) 140(5):537–46. 10.1111/j.1365-2141.2007.06965.x 18275431 PMC4477272

[10] Nuckel H Switala M Collins CH Sellmann L Grosse-Wilde H Duhrsen U High CD49d protein and mRNA expression predicts poor outcome in chronic lymphocytic leukemia. Clin Immunol (2009) 131(3):472–80. 10.1016/j.clim.2009.02.004 19318232

[11] Majid A Lin TT Best G Fishlock K Hewamana S Pratt G CD49d is an independent prognostic marker that is associated with CXCR4 expression in CLL. Leuk Res (2011) 35(6):750–6. 10.1016/j.leukres.2010.10.022 21093051

[12] Brachtl G Sahakyan K Denk U Girbl T Alinger B Hofbauer SW Differential bone marrow homing capacity of VLA-4 and CD38 high expressing chronic lymphocytic leukemia cells. PLoS One (2011) 6(8):e23758. 10.1371/journal.pone.0023758 21876768 PMC3158106

[13] Rossi D Bodoni CL Zucchetto A Rasi S De Paoli L Fangazio M Low CD49d expression and long telomere identify a chronic lymphocytic leukemia subset with highly favourable outcome. Am J Hematol (2010) 85(8):619–22 10.1002/ajh.21756 20578200

[14] Rossi D Zucchetto A Rossi FM Capello D Cerri M Deambrogi C CD49d expression is an independent risk factor of progressive disease in early stage chronic lymphocytic leukemia. Haematologica (2008) 93(10):1575–9. 10.3324/haematol.13103 18641015

[15] Brachtl G Hofbauer JP Greil R Hartmann TN. The pathogenic relevance of the prognostic markers CD38 and CD49d in chronic lymphocytic leukemia. Ann Hematol (2014) 93(3):361–74. 10.1007/s00277-013-1967-y 24288111 PMC4032465

[16] Zucchetto A Bomben R Dal Bo M Bulian P Benedetti D Nanni P CD49d in B-cell chronic lymphocytic leukemia: correlated expression with CD38 and prognostic relevance. Leukemia (2006) 20(3):523–5 10.1038/sj.leu.2404087 16408095

[17] Zucchetto A Vaisitti T Benedetti D Tissino E Bertagnolo V Rossi D The CD49d/CD29 complex is physically and functionally associated with CD38 in B-cell chronic lymphocytic leukemia cells. Leukemia (2012) 26(6):1301–12. 10.1038/leu.2011.369 22289918

[18] Buggins AGS Levi A Gohil S Fishlock K Patten PEM Calle Y Evidence for a macromolecular complex in poor prognosis CLL that contains CD38, CD49d, CD44 and MMP-9. Br J Haematol (2011) 154(2):216–22. 10.1111/j.1365-2141.2011.08725.x 21569005

[19] Collins RJ Verschuer LA Harmon BV Prentice RL Pope JH Kerr JFR. Spontaneous programmed death (apoptosis) of B-chronic lymphocytic-leukemia cells following their culture in vitro. Br J Haematol (1989) 71(3):343–50. 10.1111/j.1365-2141.1989.tb04290.x 2930721

[20] Advani RH Buggy JJ Sharman JP Smith SM Boyd TE Grant B Bruton tyrosine kinase inhibitor ibrutinib (PCI-32765) has significant activity in patients with relapsed/refractory B-cell malignancies. J Clin Oncol (2013) 31(1):88–94. 10.1200/JCO.2012.42.7906 23045577 PMC5505166

[21] Byrd JC Furman RR Coutre SE Flinn IW Burger JA Blum KA Targeting BTK with ibrutinib in relapsed chronic lymphocytic leukemia. N Engl J Med (2013) 369(1):32–42. 10.1056/NEJMoa1215637 23782158 PMC3772525

[22] Burger JA. Inhibiting B-cell receptor signaling pathways in chronic lymphocytic leukemia. Curr Hematol Malig Rep (2012) 7(1):26–33. 10.1007/s11899-011-0104-z 22105489

[23] de Rooij MFM Kuil A Geest CR Eldering E Chang BY Buggy JJ The clinically active BTK inhibitor PCI-32765 targets B-cell receptor- and chemokine-controlled adhesion and migration in chronic lymphocytic leukemia. Blood (2012) 119(11):2590–4. 10.1182/blood-2011-11-390989 22279054

[24] Wiestner A. BCR pathway inhibition as therapy for chronic lymphocytic leukemia and lymphoplasmacytic lymphoma. Hematology Am Soc Hematol Educ Program (2014) 2014(1):125–34. 10.1182/asheducation-2014.1.125 25696845

[25] Herman SE Mustafa RZ Gyamfi JA Pittaluga S Chang S Chang B Ibrutinib inhibits BCR and NF-κB signaling and reduces tumor proliferation in tissue-resident cells of patients with CLL. Blood (2014) 123(21):3286–95. 10.1182/blood-2014-02-548610 24659631 PMC4046423

[26] O’Brien S Furman RR Coutre SE Sharman JP Burger JA Blum KA Ibrutinib as initial therapy for elderly patients with chronic lymphocytic leukaemia or small lymphocytic lymphoma: an open-label, multicentre, phase 1b/2 trial. Lancet Oncol (2014) 15(1):48–58 10.1016/S1470-2045(13)70513-8 24332241 PMC4134524

[27] Furman RR Byrd JC Brown JR Coutre SE Benson DM Wagner-Johnston ND CAL-101, an isoform-selective inhibitor of phosphatidylinositol 3-kinase P110 delta, demonstrates clinical activity and pharmacodynamic effects in patients with relapsed or refractory chronic lymphocytic leukemia. Blood (ASH Annual Meeting Abstracts) (2010) 116:55.

[28] Naor D Nedvetzki S Golan I Melnik L Faitelson Y CD44 in cancer. Crit Rev Clin Lab Sci (2002) 39(6):527–79 10.1080/10408360290795574 12484499

[29] Girbl T Hinterseer E Grossinger EM Asslaber D Oberascher K Weiss L CD40-mediated activation of chronic lymphocytic leukemia cells promotes their CD44-dependent adhesion to hyaluronan and restricts CCL21-induced motility. Cancer Res (2013) 73(2):561–70. 10.1158/0008-5472.CAN-12-2749 23117883

[30] Redondo-Munoz J Ugarte-Berzal E Garcia-Marco JA del Cerro MH Van den Steen PE Opdenakker G Alpha4beta1 integrin and 190-kDa CD44v constitute a cell surface docking complex for gelatinase B/MMP-9 in chronic leukemic but not in normal B cells. Blood (2008) 112(1):169–78. 10.1182/blood-2007-08-109249 18326820

[31] DeRossi G Zarcone D Mauro F Cerruti G Tenca C Puccetti A Adhesion molecule expression on B-cell chronic lymphocytic-leukemia cells – malignant-cell phenotypes define distinct disease subsets. Blood (1993) 81(10):2679–87. 7683926

[32] Pangalis GA Roussou PA Kittas C Mitsoulismentzikoff C Matsoukaalexandridis P Anagnostopoulos N Patterns of bone-marrow involvement in chronic lymphocytic-leukemia and small lymphocytic (well differentiated) non-Hodgkins lymphoma – its clinical-significance in relation to their differential-diagnosis and prognosis. Cancer (1984) 54(4):702–8 10.1002/1097-0142(1984)54:4<702::AID-CNCR2820540418>3.0.CO;2-U 6744204

[33] Rozman C Montserrat E Rodriguezfernandez JM Avats R Vallespi T Parody R Bone-marrow histologic pattern – the best single prognostic parameter in chronic lymphocytic-leukemia – a multivariate survival analysis of 329 cases. Blood (1984) 64(3):642–8. 6466871

[34] DeRossi G Tenca C Cerruti G Favre A Zarcone D Tabilio A Adhesion molecule expression on B-cells from acute and chronic lymphoid leukemias. Leuk Lymphoma (1994) 16(1–2):31–6. 10.3109/10428199409114137 7696929

[35] Eisterer W Hilbe W Stauder R Bechter O Fend F Thaler J. An aggressive subtype of B-CLL is characterized by strong CD44 expression and lack of CD11c. Br J Haematol (1996) 93(3):661–9. 10.1046/j.1365-2141.1996.d01-1704.x 8652389

[36] Herishanu Y Gibellini F Njuguna N Hazan-Halevy I Farooqui M Bern S Activation of CD44, a receptor for extracellular matrix components, protects chronic lymphocytic leukemia cells from spontaneous and drug induced apoptosis through MCL-1. Leuk Lymphoma (2011) 52(9):1758–69. 10.3109/10428194.2011.569962 21649540 PMC3403533

[37] Fedorchenko O Stiefelhagen M Peer-Zada AA Barthel R Mayer P Eckei L CD44 regulates the apoptotic response and promotes disease development in chronic lymphocytic leukemia. Blood (2013) 121(20):4126–36. 10.1182/blood-2012-11-466250 23547049

[38] DeRossi G Marroni P Paganuzzi M Mauro FR Tenca C Zarcone D Increased serum levels of soluble CD44 standard but not of variant isoforms v5 and v6, in B cell chronic lymphocytic leukemia. Leukemia (1997) 11(1):134–41. 10.1038/sj.leu.2400525 9001429

[39] Zarcone D De Rossi G Tenca C Marroni P Mauro FR Cerruti GM Functional and clinical relevance of CD44 variant isoform expression on B-cell chronic lymphocytic leukemia cells. Haematologica (1998) 83(12):1088–98. 9949626

[40] Bazil V Horejsi V. Shedding of the Cd44 adhesion molecule from leukocytes induced by anti-Cd44 monoclonal-antibody simulating the effect of a natural receptor ligand. J Immunol (1992) 149(3):747–53. 1634766

[41] Yang H Binns RM. Isolation and characterization of the soluble and membrane-bound porcine Cd44 molecules. Immunology (1993) 78(4):547–54. 8495972 PMC1421898

[42] Ristamaki R Joensuu H GronVirta K Salmi M Jalkanen S. Origin and function of circulating CD44 in non-Hodgkin’s lymphoma. J Immunol (1997) 158(6):3000–8. 9058839

[43] Molica S Vitelli G Levato D Giannarelli D Gandolfo GM. Elevated serum levels of soluble CD44 can identify a subgroup of patients with early B-cell chronic lymphocytic leukemia who are at high risk of disease progression. Cancer (2001) 92(4):713–9. 10.1002/1097-0142(20010815)92:4<713::AID-CNCR1374>3.0.CO;2-O 11550139

[44] Eisterer W Bechter O Soderberg O Nilsson K Terol M Greil R Elevated levels of soluble CD44 are associated with advanced disease and in vitro proliferation of neoplastic lymphocytes in B-cell chronic lymphocytic leukaemia. Leuk Res (2004) 28(10):1043–51. 10.1016/j.leukres.2004.01.016 15289016

[45] Giannopoulos K Li L Bojarska-Junak A Rolinski J Dmoszynska A Hus I Expression of RHAMM/CD168 and other tumor-associated antigens in patients with B-cell chronic lymphocytic leukemia. Int J Oncol (2006) 29(1):95–103. 10.3892/ijo.29.1.95 16773189

[46] Giannopoulos K Mertens D Buhler A Barth TFE Idler I Moller P The candidate immunotherapeutical target, the receptor for hyaluronic acid-mediated motility, is associated with proliferation and shows prognostic value in B-cell chronic lymphocytic leukemia. Leukemia (2009) 23(3):519–27. 10.1038/leu.2008.338 19092852

[47] Picker LJ Toyos JD Telen MJ Haynes BF Butcher EC. Monoclonal-antibodies against the Cd44 [In(Lu)-related P80], and Pgp-1 antigens in man recognize the hermes class of lymphocyte homing receptors. J Immunol (1989) 142(6):2046–51. 2646376

[48] Jalkanen S Reichert RA Gallatin WM Bargatze RF Weissman IL Butcher EC Homing receptors and the control of lymphocyte migration. Immunol Rev (1986) 91:39–60 10.1111/j.1600-065X.1986.tb01483.x 2426181

[49] Jalkanen ST Bargatze RF Herron LR Butcher EC. A lymphoid-cell surface glycoprotein involved in endothelial-cell recognition and lymphocyte homing in man. Eur J Immunol (1986) 16(10):1195–202. 10.1002/eji.1830161003 2429846

[50] Lapidot T Dar A Kollet O. How do stem cells find their way home? Blood (2005) 106(6):1901–10. 10.1182/blood-2005-04-1417 15890683

[51] Khaldoyanidi S Denzel A Zoller M. Requirement for CD44 in proliferation and homing of hematopoietic precursor cells. J Leukoc Biol (1996) 60(5):579–92. 8929548 10.1002/jlb.60.5.579

[52] Vermeulen M Le Pesteur F Gagnerault MC Mary JY Sainteny F Lepault F. Role of adhesion molecules in the homing and mobilization of murine hematopoietic stem and progenitor cells. Blood (1998) 92(3):894–900. 9680357

[53] Avigdor A Goichberg P Shivtiel S Dar A Peled A Samira S CD44 and hyaluronic acid cooperate with SDF-1 in the trafficking of human CD34(+) stem/progenitor cells to bone marrow. Blood (2004) 103(8):2981–9. 10.1182/blood-2003-10-3611 15070674

[54] Singh V Erb U Zoller M. Cooperativity of CD44 and CD49d in leukemia cell homing, migration, and survival offers a means for therapeutic attack. J Immunol (2013) 191(10):5304–16. 10.4049/jimmunol.1301543 24127558

[55] Krause DS Lazarides K von Andrian UH Van Etten RA. Requirement for CD44 in homing and engraftment of BCR-ABL-expressing leukemic stem cells. Nat Med (2006) 12(10):1175–80. 10.1038/nm1489 16998483

[56] Jin LQ Hope KJ Zhai QL Smadja-Joffe F Dick JE. Targeting of CD44 eradicates human acute myeloid leukemic stem cells. Nat Med (2006) 12(10):1167–74. 10.1038/nm1483 16998484

[57] Asosingh K Gunthert U De Raeve H Van Riet I Van Camp B Vanderkerken K. A unique pathway in the homing of murine multiple myeloma cells: CD44v10 mediates binding to bone marrow endothelium. Cancer Res (2001) 61(7):2862–5. 11306459

[58] Hartmann TN Grabovsky V Wang W Desch P Rubenzer G Wollner S Circulating B-cell chronic lymphocytic leukemia cells display impaired migration to lymph nodes and bone marrow. Cancer Res (2009) 69(7):3121–30. 10.1158/0008-5472.CAN-08-4136 19293181

[59] Thankamony SP Sackstein R. Enforced hematopoietic cell E- and L-selectin ligand (HCELL) expression primes transendothelial migration of human mesenchymal stem cells. Proc Natl Acad Sci U S A (2011) 108(6):2258–63. 10.1073/pnas.1018064108 21257905 PMC3038774

[60] Redondo-Munoz J Ugarte-Berzal E Terol MJ Van den Steen PE del Cerro MH Roderfeld M Matrix metalloproteinase-9 promotes chronic lymphocytic leukemia B cell survival through its hemopexin domain. Cancer Cell (2010) 17(2):160–72. 10.1016/j.ccr.2009.12.044 20159608

[61] Fridman R Toth M Chvyrkova I Meroueh SO Mobashery S. Cell surface association of matrix metalloproteinase-9 (gelatinase B). Cancer and Metastasis Rev (2003) 22(2–3):153–66. 10.1023/A:1023091214123 12784994

[62] Bailon E Ugarte-Berzal E Amigo-Jimenez I Van den Steen P Opdenakker G Garcia-Marco JA Overexpression of progelatinase B/proMMP-9 affects migration regulatory pathways and impairs chronic lymphocytic leukemia cell homing to bone marrow and spleen. J Leukoc Biol (2014) 96(2):185–99 10.1189/jlb.3HI0913-521R 25080557

[63] Till KJ Zuzel M Cawley JC. The role of hyaluronan and interleukin 8 in the migration of chronic lymphocytic leukemia cells within lymphoreticular tissues. Cancer Res (1999) 59(17):4419–26. 10485492

[64] Pedersen IM Kitada S Leoni LM Zapata JM Karras JG Tsukada N Protection of CLL B cells by a follicular dendritic cell line is dependent on induction of Mcl-1. Blood (2002) 100(5):1795–801. 12176902

[65] Hamilton E Pearce L Morgan L Robinson S Ware V Brennan P Mimicking the tumour microenvironment: three different co-culture systems induce a similar phenotype but distinct proliferative signals in primary chronic lymphocytic leukaemia cells. Br J Haematol (2012) 158(5):589–99. 10.1111/j.1365-2141.2012.09191.x 22712573

[66] Hofbauer SW Krenn PW Ganghammer S Asslaber D Pichler U Oberascher K Tiam1/Rac1 signals contribute to the proliferation and chemoresistance, but not motility, of chronic lymphocytic leukemia cells. Blood (2014) 123(14):2181–8. 10.1182/blood-2013-08-523563 24501217

[67] Furman RR Asgary Z Mascarenhas JO Liou HC Schattner EJ. Modulation of NF-kappa B activity and apoptosis in chronic lymphocytic leukemia B cells. J Immunol (2000) 164(4):2200–6. 10.4049/jimmunol.164.4.2200 10657675

[68] Granziero L Ghia P Circosta P Gottardi D Strola G Geuna M Survivin is expressed on CD40 stimulation and interfaces proliferation and apoptosis in B-cell chronic lymphocytic leukemia. Blood (2001) 97(9):2777–83. 10.1182/blood.V97.9.2777 11313271

[69] Herishanu Y Perez-Galan P Liu DL Biancotto A Pittaluga S Vire B The lymph node microenvironment promotes B-cell receptor signaling, NF-kappa B activation, and tumor proliferation in chronic lymphocytic leukemia. Blood (2011) 117(2):563–74. 10.1182/blood-2010-05-284984 20940416 PMC3031480

[70] Zhang SP Wu C Farrah-Fecteau J Cul B Chen LG Zhang L Targeting chronic lymphocytic leukemia cells with a humanized monoclonal antibody specific for CD44. Proc Natl Acad Sci USA (2013) 110(15):6127–32. 10.1073/pnas.1221841110 23530247 PMC3625269

[71] Bichi R Shinton SA Martin ES Koval A Calin GA Cesari R Human chronic lymphocytic leukemia modeled in mouse by targeted TCL1 expression. Proc Natl Acad Sci U S A (2002) 99(10):6955–60. 10.1073/pnas.102181599 12011454 PMC124510

[72] Hofbauer JP Heyder C Denk U Kocher T Holler C Trapin D Development of CLL in the TCL1 transgenic mouse model is associated with severe skewing of the T-cell compartment homologous to human CLL. Leukemia (2011) 25(9):1452–8. 10.1038/leu.2011.111 21606964

[73] Choi MY Kipps TJ. Inhibitors of B-cell receptor signaling for patients with B-cell malignancies. Cancer J (2012) 18(5):404–10. 10.1097/PPO.0b013e31826c5810 23006944 PMC3461329

[74] Petlickovski A Laurenti L Li XP Marietti S Chiusolo P Sica S Sustained signaling through the B-cell receptor induces Mcl-1 and promotes survival of chronic lymphocytic leukemia B cells. Blood (2005) 105(12):4820–7. 10.1182/blood-2004-07-2669 15728130

[75] Orian-Rousseau V. CD44, a therapeutic target for metastasising tumours. Eur J Cancer (2010) 46(7):1271–7. 10.1016/j.ejca.2010.02.024 20303742

[76] Koppe M van Schaijk F Roos J van Leeuwen P Heider KH Kuthan H Safety, pharmacokinetics, immunogenicity, and biodistribution of Re-186-labeled humanized monoclonal antibody BIWA 4 (bivatuzumab) in patients with early-stage breast cancer. Cancer Biother Radiopharm (2004) 19(6):720–9. 10.1089/cbr.2004.19.720 15665619

[77] Riechelmann H Sauter A Golze W Hanft G Schroen C Hoermann K Phase I trial with the CD44v6-targeting immunoconjugate bivatuzumab mertansine in head and neck squamous cell carcinoma. Oral Oncol (2008) 44(9):823–9. 10.1016/j.oraloncology.2007.10.009 18203652

[78] Guo YJ Mazar AP Lebrun JJ Rabbani SA. An antiangiogenic urokinase-derived peptide combined with tamoxifen decreases tumor growth and metastasis in a syngeneic model of breast cancer. Cancer Res (2002) 62(16):4678–84. 12183425

[79] Piotrowicz RS Damaj BB Hachicha M Incardona F Howell SB Finlayson M. A6 peptide activates CD44 adhesive activity, induces FAK and MEK phosphorylation, and inhibits the migration and metastasis of CD44-expressing cells. Mol Cancer Ther (2011) 10(11):2072–82. 10.1158/1535-7163.MCT-11-0351 21885863

[80] Teriete P Banerji S Noble M Blundell CD Wright AJ Pickford AR Structure of the regulatory hyaluronan binding domain in the inflammatory leukocyte homing receptor CD44. Mol Cell (2004) 13(4):483–96. 10.1016/S1097-2765(04)00080-2 14992719

[81] Goodfellow PN Banting G Wiles MV Tunnacliffe A Parkar M Solomon E The gene, Mic4, which controls expression of the antigen defined by monoclonal-antibody F10.44.2, is on human chromosome-11. Eur J Immunol (1982) 12(8):659–63. 10.1002/eji.1830120807 7140811

[82] Boyd DD Kim SJ Wang H Jones TR Gallick GE A urokinase-derived peptide (angstrom 6) increases survival of mice bearing orthotopically grown prostate cancer and reduces lymph node metastasis. Am J Pathol (2003) 162(2):619–26 10.1016/S0002-9440(10)63855-2 12547719 PMC1851141

[83] van Troostenburg AR Lee D Jones TR Dyck-Jones JA Silverman MH Lam GN Safety, tolerability and pharmacokinetics of subcutaneous A6, an 8-amino acid peptide with anti-angiogenic properties, in healthy men. Int J Clin Pharmacol Ther (2004) 42(5):253–9. 10.5414/CPP42253 15176647

[84] Berkenblit A Matulonis UA Kroener JF Dezube BJ Lam GN Cuasay LC Angstrom 6, a urokinase plasminogen activator (uPA)-derived peptide in patients with advanced gynecologic cancer: a phase I trial. Gynecol Oncol (2005) 99(1):50–7. 10.1016/j.ygyno.2005.05.023 16023182

[85] Ghamande SA Silverman MH Huh W Behbakht K Ball G Cuasay L A phase 2, randomized, double-blind, placebo-controlled trial of clinical activity and safety of subcutaneous angstrom 6 in women with asymptomatic CA125 progression after first-line chemotherapy of epithelial ovarian cancer. Gynecol Oncol (2008) 111(1):89–94 10.1016/j.ygyno.2008.06.028 18760451

[86] Weigand S Herting F Maisel D Nopora A Voss E Schaab C Global quantitative phosphoproteome analysis of human tumor xenografts treated with a CD44 antagonist. Cancer Res (2012) 72(17):4329–39. 10.1158/0008-5472.CAN-12-0136 22777824

